# Host–MicroRNA–Microbiota Interactions in Colorectal Cancer

**DOI:** 10.3390/genes10040270

**Published:** 2019-04-02

**Authors:** Ce Yuan, Clifford J. Steer, Subbaya Subramanian

**Affiliations:** 1 Bioinformatics and Computational Biology Program, University of Minnesota, Minneapolis, MN 55455, USA; yuanx236@umn.edu; 2 Department of Surgery, University of Minnesota Medical School, Minneapolis, MN 55455, USA; 3 Department of Medicine, University of Minnesota Medical School, Minneapolis, MN 55455, USA; steer001@umn.edu; 4 Department of Genetics, Cell Biology, and Development, University of Minnesota Medical School, Minneapolis, MN 55455, USA

**Keywords:** colorectal cancer, microRNAs, gut microbiota, metabolic interactions

## Abstract

Changes in gut microbiota composition have consistently been observed in patients with colorectal cancer (CRC). Yet, it is not entirely clear how the gut microbiota interacts with tumor cells. We know that tumor cells undergo a drastic change in energy metabolism, mediated by microRNAs (miRNAs), and that tumor-derived miRNAs affect the stromal and immune cell fractions of the tumor microenvironment. Recent studies suggest that host intestinal miRNAs can also affect the growth and composition of the gut microbiota. Our previous CRC studies showed a high-level of interconnectedness between host miRNAs and their microbiota. Considering all the evidence to date, we postulate that the altered nutrient composition and miRNA expression in the CRC microenvironment selectively exerts pressure on the surrounding microbiota, leading to alterations in its composition. In this review article, we present our current understanding of the role of miRNAs in mediating host–microbiota interactions in CRC.

## 1. Introduction

An average human intestine contains more than 100 trillion bacteria (collectively known as the gut microbiota) [1]. In recent decades, a number of studies have suggested that the gut microbiota is crucial to human health and to the development of diseases, including colorectal cancer (CRC) [2,3,4,5,6,7]. Those studies determined that altered microbiota composition and function (dysbiosis) is a common signature of CRC. Bacterial candidates such as *Fusobacterium nucleatum* and *Bacteroides fragilis* are consistently enriched in tumor tissues, and included in that signature. Specific factors in those bacteria, including FadA and Fap2 protein from *F. nucleatum* and *B. fragilis* toxins, that play a role in CRC pathobiology have been identified [8,9,10,11,12,13,14,15,16]. However, our knowledge of the vast majority of other bacteria associated with the CRC microenvironment is limited. Moreover, we are just beginning to understand the complex interactions between host and microbiota in CRC, as well as other clinical disorders including neurodegenerative diseases [17].

In healthy humans, a key factor associated with microbiota variations is host genetics [18,19,20,21]. In a study of healthy twins, Goodrich et al. found that host genetics drive microbiota composition and can also affect the host metabolic phenotype [19]. Several other studies have found an association between the abundance of *Bifidobacterium* species and the presence of single-nucleotide polymorphisms (SNPs) in close proximity to the host lactase gene locus [18,22]. This association suggests that the *Bifidobacterium* species conceivably assists the host in metabolizing lactose.

A recent CRC study found that loss-of-function mutations in the mitogen-activated protein kinase (MAPK) and Wnt signaling pathways are associated with specific sets of microbiota profiles [2]. Furthermore, mutations in the tumor-suppressor adenomatous polyposis coli (APC) gene are also associated with a distinct inter-microbiota association network [2]. These findings suggest that a common genetic factor might orchestrate the dynamic host–microbiota interaction(s) and functional relationship(s). Indeed, other recent studies have provided experimental evidence that microRNAs (miRNAs) can influence the survival and composition of gut bacteria [3,23,24]. Moreover, miRNAs have important intermediate roles in regulating CRC transformation and progression via the action of signaling pathways, including MAPK, Wnt, and APC [25,26]. MicroRNAs are small noncoding RNAs (about 22 nt) that play an important role in regulating and fine-tuning gene expression [27]. In mammalian cells, miRNAs regulate gene expression through posttranscriptional modifications in two distinct, albeit paired, mechanisms. First, if the miRNA has an extensive complementary binding site in the messenger RNA (mRNA) target, then it will guide the RNA-induced silencing complex (RISC) to cleave the mRNA, thus inhibiting translation. Second, if the miRNA only partially binds to the 3’ untranslated region (3’UTR) of the mRNA, then the miRNA-RISC will act to repress mRNA translation [28]. Both mechanisms lead to the decreased translation of mRNAs, which alters their respective downstream functions. Because miRNAs can act upon mRNA targets with limited complementarity, each miRNA can target a wide range of mRNAs in mammalian cells and each mRNA can be targeted by numerous miRNAs. More than 30% of human genes are estimated to have conserved binding sites in the 3’UTR [29]. Clearly, given this vast and enormously complex regulatory network, miRNAs are immensely important in regulating critical cellular processes. We are only now beginning to understand the sophisticated cross-talk of miRNAs, not only with each other, but with the myriad of target mRNAs.

**Figure 1:**
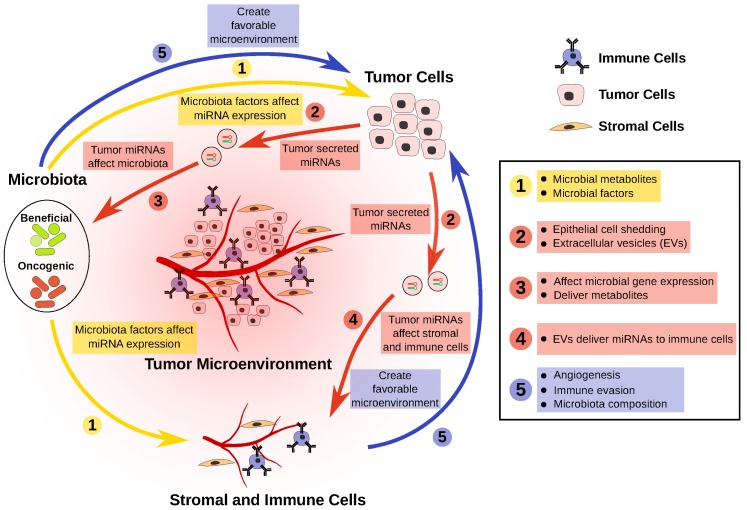
Host–microRNA–microbiota interactions in colorectal cancer. Microbiota composition has a functional effect on the cancer cells, via stromal and tumor infiltrating immune cells by regulating various cellular process (**1**). Microbial-metabolites and other secreted factors affect miRNA/gene expression profiles in cells present in the tumor microenvironment. In turn, tumor cells affect the microbiota composition of the stromal and tumor infiltrating immune cells through shedding of epithelial cells and/or secreting extracellular vesicles (EVs) containing miRNAs (**2**). The tumor-miRNAs alter the microbiota composition by affecting the gene expression of the microbiota and by delivering cancer-secreted metabolites (**3**). The tumor-derived miRNAs also have a role in regulating stromal and tumor infiltrating immune cells by affecting gene expression through miRNAs delivered in EVs (**4**). Such interactions will finally create a favorable microenvironment for tumor cells that include angiogenesis, immune evasion, and microbiota composition (**5**).

Based on mounting evidence, we postulate that the altered nutrient composition and miRNA expression in the CRC microenvironment selectively influences the surrounding microbiota, leading to alterations in its composition. In this review, we present our current understanding of the role of miRNAs in mediating host–microbiota interactions in CRC (Figure 1). After highlighting the evidence pointing to their central role, we reflect on the future direction of this rapidly evolving field.

## 2. Microbiota and Colorectal Cancer

In the healthy intestine, the microbiota maintains a stable structure and actively participates in energy harvesting and nutrient production from undigested food [30,31]. However, this balance is disrupted in patients with CRC. Current evidence suggests that the microbiota regulates host functions via both metabolites and secreted factors.

### 2.1. Microbial Metabolites

In a normal colon, the microbiota produces a vast number of metabolites. Some of them, including vitamin K, biotin, and short-chain fatty acids (SCFAs), are essential for maintaining homeostasis in the colon microenvironment [31]. In fact, the major energy source (∼70%) required by colon epithelium is butyrate, which is produced by the microbiota through fermentation of complex carbohydrates. Without the microbiota, the colon epithelium undergoes autophagy and fails to maintain its normal structure and function [32]. Similarly, mice lacking a microbiota (i.e., germ-free mice or those treated by broad-spectrum antibiotics) develop significantly fewer tumors in the colon [33,34,35]. However, in humans, using broad-spectrum antibiotics to treat CRC is not feasible, because of the risk of introducing harmful and highly resistant secondary infections such as *Clostridium difficile*.

In our current understanding, a few main classes of bacterial metabolites play a key role in the pathogenesis of CRC and the immune microenvironment. These metabolites include SCFAs, polyamines, secondary bile acids, and phytochemicals. Their role in CRC has been extensively reviewed and documented [31,36,37,38]. We have recently also explored the role of miRNAs in mediating the effect of microbial metabolites on CRC and its microenvironment [39].

### 2.2. Microbial Factors

Early studies consistently found a greater population of *F. nucleatum* in microbiota samples in patients with CRC than in healthy controls [40,41]. This bacterium is commonly found in the human oral microbiota and is frequently associated with gum diseases; it is, however, not commonly present in the gut microbiota. Through the Fap2 virulence factor, it uniquely binds with the D-galactose-β(1-3)-N-acetyl-D-galactosamine (Gal-GalNAc) carbohydrate moiety expressed on the tumor surface of CRCs [14]. Once it localizes to the CRC microenvironment, it targets the Wnt/β-catenin signaling pathway by binding, via association with the FadA virulence factor, to the E-cadherin protein on the cell surface [16]. The Wnt/β-catenin signaling pathway is critical during tumor initiation, tumor migration, and metabolic reprogramming [42,43,44,45]. The role of the Wnt/β-catenin signaling pathway in CRC has been previously reviewed [45].

Another bacterial protein targeting the same Wnt/β-catenin signaling pathway is the Bacteroides fragilis toxin (bft) produced by *B. fragilis* [46]. The bft virulence factor is able to bind to the E-cadherin protein, similar to that of FadA, but additionally cleaves the protein, which can alter the intestinal tight-junction function [47]. The Wnt/β-catenin pathway is a major signaling pathway that controls the expression of many important tumor-related genes, including MYC. The transcription factor MYC, transactivates miRNAs, such as the miR-17-92 cluster, that are highly expressed in CRC [48,49,50,51].

Additionally, *F. nucleatum* can also induce CRC cell proliferation by upregulating miR-21, via activation of the nuclear factor kappa-light-chain-enhancer of activated B cells (NF-κB) pathway via toll-like receptor 4 (TLR4) signaling [52]. The *Escherichia coli* bacterium harboring the pks genomic island also plays an important role in CRC. When CRC cells come in contact with the colibactin genotoxin produced by *E. coli*, the cells undergo cellular senescence [53,54]. This process is mediated by the cellular upregulation of miR-20a-5p, which results in the downregulation of sentrin-specific protease 1 (SENP1). This process then alters p53 small ubiquitin-like modifier (SUMO)ylation, which has been shown to affect the growth and metastasis of tumor cells [55].

In addition to factors that are virulent, many bacteria also produce beneficial factors that can reduce inflammation and modulate the immune system. In germ-free mice, early studies found impaired intestinal immune systems, which were amenable to treatment [56]. Specifically, the *B. fragilis* polysaccharide A (PSA) is one such immunomodulatory factor that maintains the proper function of CD4+ T cells [57]. Several other polysaccharides produced by *B. fragilis* are also beneficial in maintaining proper immune function. Immunization with *B. fragilis* polysaccharides, or the adoptive transfer of T cells specific to *B. fragilis*, can even boost the treatment effect of anti-cytotoxic T-lymphocyte antigen 4 (CTLA-4) immunotherapy [58]. The seemingly conflicting role of *B. fragilis* within gut bacteria is only the tip of the iceberg in current microbiota research and the fine and highly complex balance between functions.

In light of all this evidence, we created the first system-level map of interactions between host miRNAs and the microbiota [3]. Our comprehensive map helped us analyze correlations between host miRNA expression levels and mucosa-associated microbiota profiles, specifically in patients with CRC. 

## 3. MicroRNAs and Colorectal Cancer

Previous studies have identified numerous aberrant miRNA expression patterns in CRC [25,59,60,61,62]. Specifically, the miR-17-92 cluster, miR-21, miR-182, and miR-503 are consistently overexpressed in tumor (vs. normal) tissues [3,26,48,49,59,63,64,65,66,67,68,69,70,71]. Any alteration(s) in expression levels of these miRNAs could, in turn, affect a wide array of downstream gene targets. Together, these miRNAs regulate all aspects of tumor pathobiology, including (i) altering tumor metabolism; (ii) promoting cell proliferation; (iii) stimulating angiogenesis; (iv) down-regulating tumor-suppressor genes; (v) promoting evasion of immune surveillance; and (vi) creating a favorable tumor microenvironment that promotes invasion and metastasis.

Our laboratory previously reported that, during the adenoma to adenocarcinoma transition, miR-182 and miR-503 were sequentially overexpressed and targeted the tumor-suppressor *FBXW7* gene [69]. Other researchers have observed, during CRC transformation, an increased expression of the miR-17-92 cluster and miR-21 [48,72]. In CRC adenocarcinoma, members of the miR-17-92 cluster target transforming growth factor-beta (TGF-β), which in turn stimulates angiogenesis in the tumor microenvironment, thus promoting tumor growth [70]. Additionally, miR-19, a member of the miR-17-92 cluster, downregulates expression of the tumor-suppressor phosphatase and tensin homolog (PTEN), thereby activating the protein kinase B (AKT)/mammalian target of rapamycin (mTOR) pathway in tumor cells [73]. The AKT/mTOR pathway is the main metabolic sensing pathway, responsible for regulating glucose transport into cells [74]. Since glucose is the main fuel source of CRC cells, an activated AKT/mTOR pathway promotes tumor cell proliferation [75].

The tumor-suppressor *PDCD4* gene, which is commonly downregulated in CRC, is a target of miR-21 [67]. Inhibiting the *PDCD4* gene can lead to an increase in the metastasis potential of tumor cells. Another important pathway commonly altered in CRC tumors is the Wnt/β-catenin pathway [25,26]. Dozens of miRNAs have been shown to extensively regulate the genes involved in the Wnt/β-catenin pathway [25]. 

The complex microenvironment of the CRC tumor also involves stromal cell and immune cell fractions, which can be regulated by cancer-derived miRNAs [76,77,78]. Studies have found that the miR-17-92 cluster, commonly overexpressed in CRC cells, is also upregulated in CRC stromal cells [68,72,79]. Strikingly, these miRNAs are not only endogenously produced by stromal cells, but also packaged in the microvesicles of tumor cells, and then delivered to stromal cells [80,81]. Similar intracellular regulation mediated by miRNAs is also found in immune cell fractions [82]. Additionally, endogenous miRNA dysregulation is prevalent in CRC immune cell fractions, usually as a downstream effect of tumor-secreted factors such as cytokines and chemokines [83,84,85]. Collectively, this evidence suggests that miRNAs are important in regulating tumor cells, in addition to maintaining the tumor microenvironment. It is clear that the relationship between miRNAs and CRC is multifaceted, interrelated, and highly complex.

## 4. Host Regulation of Microbiota Mediated by MicroRNAs

In reestablishing germ-free mice with a normal microbiota, studies have found altered intestinal miRNA profiles, suggesting that the microbiota regulates host miRNA expression [86,87]. Moreover, the responses of intestinal cells to facilitating the microbiota process depends on the cell type, and intestinal epithelial stem cells are especially sensitive to microbiota reestablishment [87].

Because miRNAs are highly stable, several studies in the clinical arena were able to detect higher levels of miR-21 and miR-92a, among other miRNAs, in the fecal samples of patients with CRC [65,88,89]. This finding facilitated in developing a noninvasive CRC screening method and delineating the potential role of miRNAs in interacting with the trillions of microbes in the human gut.

Intestinal miRNAs develop from two main sources, including the host and the food [23,24]. The intestinal epithelial cells are the main contributors of host-derived miRNAs, either via shedding of cells or excretion of exosomes. Evidence has shown that miRNAs from food can be absorbed by the host and can affect host gene expression [90,91,92]. But certain food-sourced miRNAs remain stable in the digestive tract and reach the intestines [93,94]. This evidence suggests that miRNAs can mediate cross-species regulation. The idea remains nascent, so insight into how miRNAs mediate host–microbiota interactions is still limited. Liu et al. first demonstrated such regulation, showing that miRNAs present in the feces can regulate gene expression and growth of bacteria [23]. Specifically, they found that mice lacking the Dicer gene, which enables mature miRNA processing, had different microbiota profiles than wild-type mice. More importantly, the study reported that hsa-miR-515-5p promoted the growth of *F. nucleatum* in vitro by targeting the 16S ribosomal RNA (rRNA) gene.

Notably, however, hsa-miR-515-5p shows very low expression levels in CRC tumors, so they are not significantly different from normal tissue. Thus, interactions between hsa-miR-515-5p and *F. nucleatum* might not be significant in CRC pathogenesis. However, more importantly, this study found that fecal miRNA transplantation restores fecal microbiota composition in mice with Dicer gene knockout. Several recent studies found that fecal microbiota transplantation (FMT) offers a potential therapeutic benefit that enables an immunotherapeutic response [35,95,96,97,98]. Based on growing evidence, it is plausible that fecal miRNAs play an important role in modulating the CRC microbiota as well as immunotherapy responses. 

Recently, Teng et al. demonstrated that miRNAs encapsulated in plant-derived exosome-like nanoparticles (ELNs) can enter bacteria and alter bacterial genes [24]. The process for bacterial uptake of ELNs is determined primarily by the lipid composition of the outer membrane. They found that ELNs enriched with phosphatidylcholine were preferentially taken up by the *Ruminococcus* species, whereas ELNs enriched with phosphatidic acid (PA) were primarily taken up by *Lactobacillus rhamnosus*. After the ELNs are taken up by specific bacteria, the miRNA contents are released into bacterial cells. Teng et al. also found that mdo-miR7267-3p encapsulated in the PA-enriched ELNs targets the *Lactobacillus* monooxygenase ycnE, which then increases its production of indole-3-carboxaldehyde (I3A). The I3A metabolite then promotes interleukin-22 (IL-22) production and helps repair damaged colon mucosa [99].

There is developing evidence to support the notion that host or exogenous miRNAs might be biologically active in bacteria, thereby affecting bacterial gene expression. Although small RNAs similar to miRNAs exist in bacteria and function similarly to miRNAs, it remains unknown as to how miRNAs function in bacteria [100]. Several studies have reported that exogenous miRNAs from plant or animal sources can be taken up by human cells and exert biological functions [90,91,92,93,94,101,102,103]. Additional studies are required to ascertain whether miRNAs can indeed affect bacteria and to delineate the precise mechanism(s).

## 5. Metabolic Changes in Colorectal Cancer and Microbiota Mediated by MicroRNAs

The prevailing “driver-passenger” model suggests that dysbiosis in the CRC microbiota is initially caused by colonization of driver bacteria. This is followed by a gradual change in the tumor microenvironment, an increase in the number of driver bacteria, and secondary colonization of passenger bacteria that benefit from the changed environment [104]. That model, together with other studies, suggest that a gradual metabolic change in the tumor microenvironment during cancer progression could be the cause of dysbiosis [31]. Again, we explored that issue in our recent review of the role of miRNAs in mediating the effect of microbial metabolites on CRC and its microenvironment [39].

One of the hallmarks of tumor growth is their increased use of glycolysis as a main energy source, known universally as the Warburg effect [105]. Because the normal colon uses butyrate as its major energy source, any change in that source preferred by proliferating tumor cells will undoubtedly profoundly alter the nutrient composition of the tumor microenvironment [106,107]. Indeed, several studies have found altered metabolite levels in CRC tissues and stools [107,108,109,110]. A significantly lower glucose level and higher levels of lactate and fatty acids have been found in CRC tumor tissues, as compared with adjacent normal tissues. In stool samples from patients with CRC, a higher level of amino acids and a lower level of fatty acids have also been observed [108]. Interestingly, the CRC microbiota has shown reduced carbohydrate metabolism and an increase in the biosynthesis of amino acids and fatty acids [41]. In CRC, the switch in the nutrient source preferred by proliferating tumor cells appears to alter the nutrient composition in the tumor microenvironment. At the same time, the nutrient metabolism of the tumor microbiota seems to complement the nutrient needs of the tumor. This could be due to factors associated with the tumor nutrient microenvironment, and by the miRNAs excreted by tumor cells, on the surrounding microbiota [3]. Given the role of miRNAs in mediating such metabolic changes, we believe that miRNAs play a central, if not critical role, in mediating host–microbiota metabolic interactions in CRC.

## 6. Conclusions and Perspectives

With thousands of bacterial species living in the human digestive tract, it is becoming quite evident that they profoundly affect human health. Our review of the recent literature regarding CRC underscores a complex metabolic interplay between the host and its microbiota, mediated in part by miRNAs. Based on the current literature, we offer five major points in host–microbiota interactions mediated by miRNAs (Figure 1):

1. The CRC microbiota has reduced representation of beneficial bacteria. These bacteria produce metabolites and other factors that can potentially slow CRC progression, in part via the modulation of miRNAs that regulate tumor cells.
2. Dysregulation of miRNAs in tumor cells can affect the survival, or the gene expression, of certain bacteria in the microbiota.
3. Dysregulated miRNAs in tumor cells can be packaged and delivered to both stromal and immune cell fractions, creating a more favorable microenvironment for tumor cells.
4. Overrepresentation of oncogenic bacteria in the CRC microbiota can modulate tumor cells, as well as the tumor microenvironment, through miRNA modulation, thereby resulting in a more favorable condition for tumor growth.
5. This negative feedback loop perpetuates CRC progression.

Potential methods to break such a negative feedback loop include:

1. Interfering with host-mediated microbiota modulation by designing strategies to deliver anti-miRNAs to block the effect of host-miRNAs on the microbiota.
2. Modulating the microbiota through miRNAs that promote the growth of beneficial bacteria while suppressing the growth of oncogenic bacteria, in conjunction with chemotherapy or immunotherapy.

Based on both experimental and computational data, we conclude that miRNAs mediate and critically influence host–microbiota interactions. Clearly, miRNAs are a major part of a complex web of highly dynamic interactions. Other factors, such as nutrient availability in the CRC microenvironment, could also play an important role. In the future, it will be imperative to use a combination of approaches to comprehensively survey the CRC microenvironment, in order to discover all potential players in mediating such interactions.
